# Application of Wnt Pathway Inhibitor Delivering Scaffold for Inhibiting Fibrosis in Urethra Strictures: *In Vitro* and *in Vivo* Study

**DOI:** 10.3390/ijms161126050

**Published:** 2015-11-19

**Authors:** Kaile Zhang, Xuran Guo, Weixin Zhao, Guoguang Niu, Xiumei Mo, Qiang Fu

**Affiliations:** 1 The Department of Urology, Affiliated Sixth People’s Hospital, Shanghai Jiaotong University, Yishan Road 600, Shanghai 200233, China; great_z0313@126.com; 2 Biomaterials and Tissue Engineering Laboratory, College of Chemistry & Chemical Engineering and Biotechnology, Donghua University, North Renmin Road 2999, Shanghai 201620, China; ibmguoxuran@163.com; 3 Wake Forest Institute for Regenerative Medicine, 391 Technology Way, Winston-Salem, NC 27101, USA; wezhao@wakehealth.edu (W.Z.); gniu@wakehealth.edu (G.N.)

**Keywords:** Wnt signaling pathway, electrospinning, urethra, tissue engineering, regenerative medicine

## Abstract

**Objective:** To evaluate the mechanical property and biocompatibility of the Wnt pathway inhibitor (ICG-001) delivering collagen/poly(l-lactide-*co*-caprolactone) (P(LLA-CL)) scaffold for urethroplasty, and also the feasibility of inhibiting the extracellular matrix (ECM) expression *in vitro* and *in vivo*. **Methods:** ICG-001 (1 mg (2 mM)) was loaded into a (P(LLA-CL)) scaffold with the co-axial electrospinning technique. The characteristics of the mechanical property and drug release fashion of scaffolds were tested with a mechanical testing machine (Instron) and high-performance liquid chromatography (HPLC). Rabbit bladder epithelial cells and the dermal fibroblasts were isolated by enzymatic digestion method. (3-(4,5-Dimethylthiazol-2-yl)-2,5-Diphenyltetrazolium Bromide (MTT) assay) and scanning electron microscopy (SEM) were used to evaluate the viability and proliferation of the cells on the scaffolds. Fibrolasts treated with TGF-β1 and ICG-001 released medium from scaffolds were used to evaluate the anti-fibrosis effect through immunofluorescence, real time PCR and western blot. Urethrography and histology were used to evaluate the efficacy of urethral implantation. **Results:** The scaffold delivering ICG-001 was fabricated, the fiber diameter and mechanical strength of scaffolds with inhibitor were comparable with the non-drug scaffold. The SEM and MTT assay showed no toxic effect of ICG-001 to the proliferation of epithelial cells on the collagen/P(LLA-CL) scaffold with ICG-001. After treatment with culture medium released from the drug-delivering scaffold, the expression of Collagen type 1, 3 and fibronectin of fibroblasts could be inhibited significantly at the mRNA and protein levels. In the results of urethrography, urethral strictures and fistulas were found in the rabbits treated with non-ICG-001 delivering scaffolds, but all the rabbits treated with ICG-001-delivering scaffolds showed wide caliber in urethras. Histology results showed less collagen but more smooth muscle and thicker epithelium in urethras repaired with ICG-001 delivering scaffolds. **Conclusion:** After loading with the Wnt signal pathway inhibitor ICG-001, the Collagen/P(LLA-CL) scaffold could facilitate a decrease in the ECM deposition of fibroblasts. The ICG-001 delivering Collagen/P(LLA-CL) nanofibrous scaffold seeded with epithelial cells has the potential to be a promising substitute material for urethroplasty. Longer follow-up study in larger animals is needed in the future.

## 1. Introduction

Urethral strictures were common diseases after urethral injury, which seriously affect the life quality of patients [1]. It has an incidence of 0.6% in male patients and results from a variety of types of etiological factors, such as mechanical, heat, and radiation [2]. The urethral defect was often healed with extracellular matrix (ECM) over expression which inevitably caused a reduction in the urethral caliber and impairment to the flow of urine [3]. Traditionally, these defects need surgery of urethral reconstruction with a substituted material such as an autologous penile flap or oral mucosa graft. However, these autologous materials will inevitably lead to serious morbidities at the donor site like infection, nerve injury and difficulties in opening the mouth *etc.* [4].

In spite of the rapid developments of the surgical procedures for urethroplasty, the recurrence rate was still high (approximately 20%) because of submucosa fibrosis and scar formation in the urethra after substitute surgery [1,5]. In recent years, the rapid development of regenerative medicine and engineered urethras provided a new procedure for the reconstruction of urethras [6,7,8]. In our previous research, scaffold only or scaffold seeded with epithelial cells for urethral reconstruction were applied in animal models [9,10]. Satisfactory results in a short period have been acquired in previous studies, however, submucosal tissue of neonatal urethra contains more collagen fibers and aligned messily compared with the natural urethral submucosa. This phenomenon is similar to the performance of fibrosis which is the most common cause of failed tissue engineered urethra reconstruction.

Excessively deposited ECM of urethral submucosa was believed to be closely related to urethral restructure [11]. Significant graft fibrosis would thus invariably result in surgery failure and restricture in patients. In a preliminary study, we used rabbit fibroblasts for which the TGF-β gene was silenced by siRNA to repair the urethral submucosa [12,13]. These fibroblasts significantly inhibited ECM production in the rabbit urethral submucosa. However, such genetic technology was difficult to apply from bench to bedside due to ethical and technical aspects.

It is known that the TGF-β pathway plays an important part in a variety of fibrotic diseases [14,15,16], and that the urethral tissue taken from patients with urethral strictures also overexpressed TGF-β1 gene [17,18]. It was also reported that TGF-β1 injection in urethra can successfully generate a reproducible rat model of urethral spongiofibrosis [19]. Many researches showed that a canonical Wnt/β-catenin signaling pathway was a downstream regulatory pathway of the TGF-β pathway [20,21,22,23]. TGF-β stimulates canonical Wnt signaling and activation of canonical Wnt signaling contributes to the profibrotic effects of TGF-β [24]. Blocking of the Wnt signaling pathway was demonstrated to be efficient in curing fibrosis in skin, kidney, and lung, *etc.* [25,26,27,28,29,30]. To inhibit Wnt signaling instead of TGF-β signaling might therefore be a promising solution for urethral stricture but without severe adverse effects caused by blocked TGF-β signaling. Wnt inhibitors that target β-catenin/TCF interaction or β-catenin co-factor recruitment may represent potential therapeutic approaches for fibrosis [26]. Furthermore, varieties of modulators of the Wnt pathway with small molecular weight have been discovered, such as ICG-001, and PKF118-310 *etc.* The Wnt pathway inhibiter ICG-001 we used here is a small molecule with 548.63 in molecular weight [31].

Electrospinning is an adaptable method for fabrication of scaffolds [32]. The scaffolds fabricated by electrospinning exhibit high porosity and micro to nano scale topography, similar to the structure of natural ECM, and are widely used in the engineering of various tissues [33]. Here, we constructed a novel electrospun nanofiber scaffold delivering ICG-001 through the co-axial electrospinning technique. The nanofiber was composed of collagen type 1 and poly(l-lactide-*co*-caprolactone) (P(LLA-CL)), which morphologically and structurally mimicked the ECM of native tissue [34]. Collagen is an important extracellular matrix protein component, possessing a natural biocompatibility, the application of collagen can promote the expansion and differentiation of urothelial cells, however the mechanical properties of the collagen scaffold alone is relatively brittle and fragile. Therefore, a combination of materials was made in order to fully satisfy the mechanical properties of the tissue engineered urethra. The (P(LLA-CL)) is the copolymer of l-lactic acid and ε-caprolactone, which possesses good biocompatibility, biological degradability and mechanical properties, but because of its hydrophobic characteristic, it is not conducive to cell adhesion and proliferation. In our study, collagen and P(LLA-CL) were combined to obtain good biocompatibility and mechanical strength of the desired nanofiber [33]. Compared with the method of blending the drug with the polymer materials directly, the core-shell co-axial electrospinning could decrease the burst release and protect the drug activity in the process of fabrication [35,36].

In this study, We combined the technique of co-axial electrospinning technology with the Wnt pathway inhibitor ICG-001 to produce a functional electrospun collagen/P(LLA-CL) nanofiber scaffold, then evaluated its anti-fibrosis effect *in vitro* and in rabbits urethral defects model in order to provide preliminary evidence and foundation for the large animal study and clinical practice in the future.

## 2. Results and Discussion

### 2.1. Characteristics of Scaffolds

**Figure 1:**
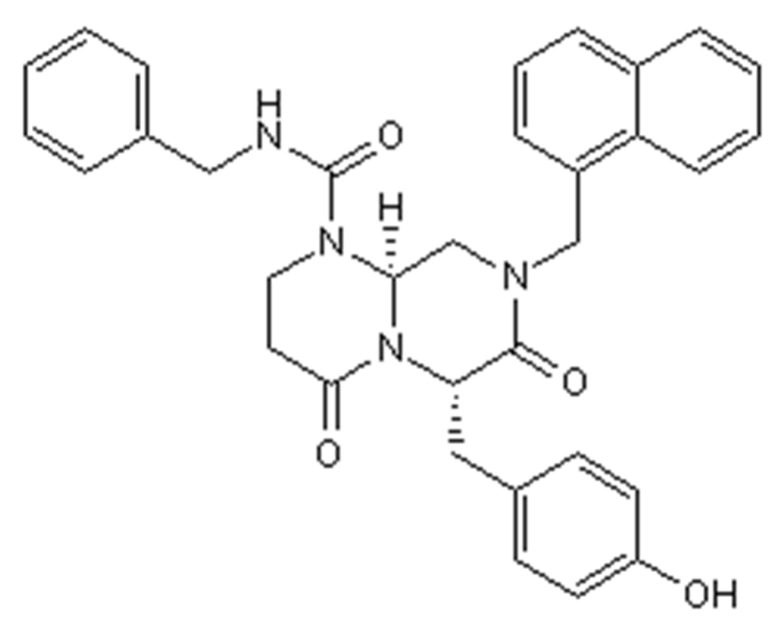
Chemical structure of ICG-001.

**Figure 2:**
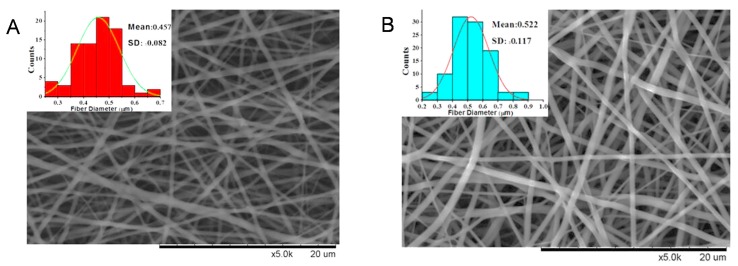

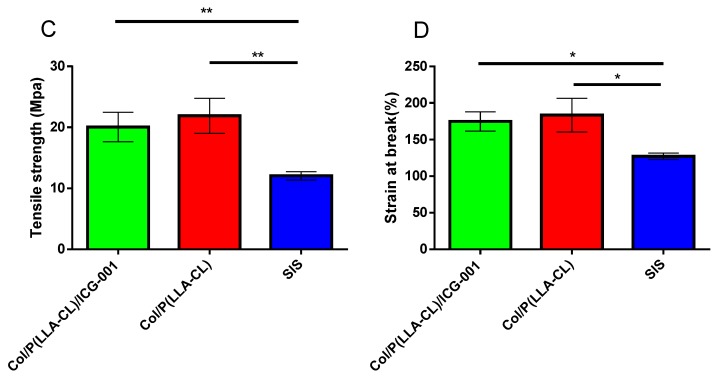
The mechanical properties of different scaffolds. (**A**) The non-drug scaffold; (**B**) The ICG-001 delivering scaffold; (**C**) Tensile strength of scaffolds and small intestinal submucosa (SIS); and (**D**) Stress at break of scaffolds and SIS. * *p* < 0.05; ** *p* < 0.01.

The thickness of the non-drug scaffolds was 0.75 ± 0.16 mm and ICG-001 (Figure 1) delivering scaffold 0.78 ± 0.12 mm. The SEM figures of the scaffolds showed that both the drug delivering fibers and non-drug delivering fibers formed a structure with high interconnection and porosity. 200 fibers of each scaffold were measured, the drug delivering fiber diameter was 457 ± 82 nm (Figure 2A) and the fiber diameter without drug delivering was 522 ± 177 nm (Figure 2B). The small intestinal submucosa (SIS, Cook medical, IN, USA), a commercial substitute material for urethroplasty, was used as a control to compare the mechanical property. The mechanical property included tensile strength (Figure 2C), strain at break (Figure 2D). Both scaffolds showed higher tensile strength and strain at break than SIS significantly. The non-drug and drug delivering scaffolds showed no significant difference.

### 2.2. In Vitro Release of ICG-001 from the Scaffolds

**Figure 3:**
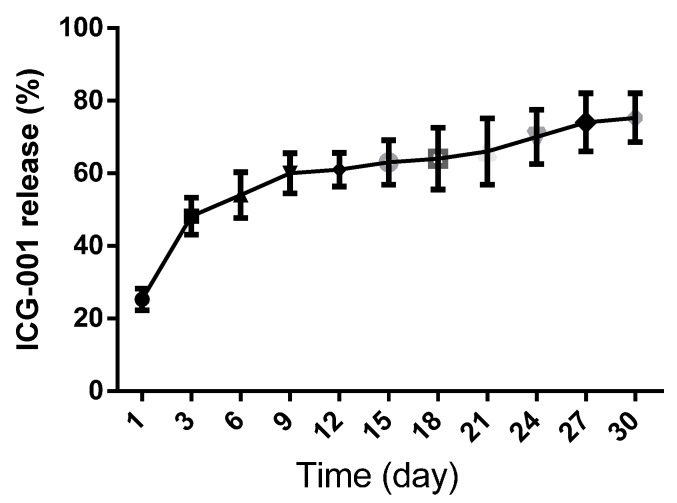
The cumulative release of the ICG-001 delivered scaffold in 30 days *in vitro*.

The controlled release of ICG-001 from drug delivering collagen/P(LLA-CL) scaffolds were analyzed with High-performance liquid chromatography (HPLC) (Figure 3). The release of ICG-001 was tested from day one after the scaffold was immerged in the PBS solution. The theoretical weight of ICG-001 in scaffold specimen is 0.01 mg according to the total area and weight of the electrospun scaffold. In the PBS, the release experienced two stages: the initial burst released before day three, and the continuous release from day three to day 30. On day three, the percentage in released solution was over 48% (0.048 mg). After day three, the release of ICG-001 was sustained and the curve showed a stable trend. The percentage reached 75% (0.075 mg) after day 30.

### 2.3. Cell Isolation and Identification

**Figure 4:**
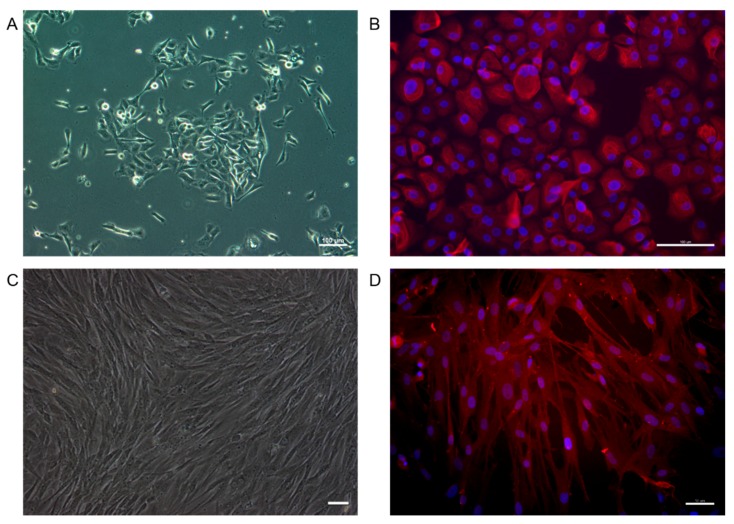
The characteristic of rabbit bladder epithelial cells was typical (**A**); The marker of epithelial cells (pan Cytokeratin) (Red) immunofluorescent staining of epithelial cells was positive (**B**); The dermal fibroblasts were isolated (**C**); (Scale bar: 50 μm) and the vimentin (Red) was identified (**D**). Blue: DAPI staining of nuclei.

Successful epithelial cell (Figure 4A) and fibroblasts (Figure 4C) cultures were obtained from biopsy of bladder and dermal tissue used in the study. Bladder epithelial cells were isolated and expanded until enough numbers of cells were obtained. The epithelial cells showed the expression of pan Cytokeratin (Figure 4B), which is a specific marker of epithelial cells. The fibroblasts were identified with vimentin (Figure 4D).

Cultured epithelial cells were seeded onto each scaffold with a density of 0.5 million/cm^2^. The structure with cells was cultured in D-KSFM for 1 week before being tested and implanted into the animal. Fibroblasts were cultured and passaged in six-well plates for the anti-fibrosis test.

**Figure 5:**
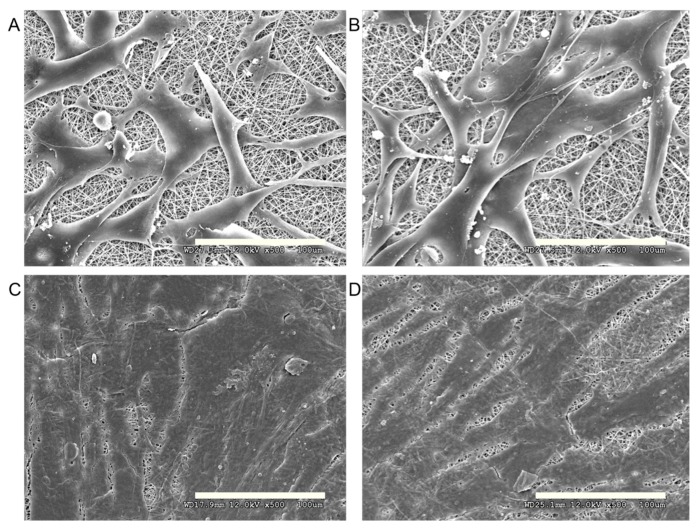
SEM image showed that epithelial cells expanded well on Col/P(LLA-CL) scaffold ((**A**) day three; (**C**) day seven) and Col/P(LLA-CL)/ICG-001 scaffold ((**B**) day three; (**D**) day seven). Scale bar: 100 μm.

### 2.4. Cell Morphology and Proliferation on Scaffold

On day three and seven, SEM was used to observe the growth of the cells on the scaffolds. The SEM showed that the epithelial cells attached well on both kinds of scaffolds on day three (Figure 5A,B). The cells stretched peripherally to the pores of the scaffold and connected to each other. The cells proliferated and expanded to cover the majority of the scaffold on day seven (Figure 5C,D).

**Figure 6:**
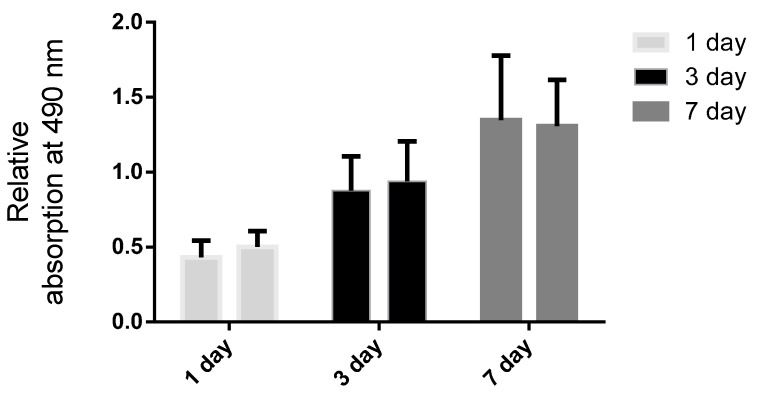
The relative absorption of the 3-(4,5-Dimethylthiazol-2-yl)-2,5-Diphenyltetrazolium Bromide (MTT) assay for epithelial cells on normal scaffold without ICG-001, and ICG-001-delivering scaffold on day one, three and seven.

MTT assay showed a similar rate of proliferation of epithelial cells on both non-drug scaffold and drug delivering scaffold. The proliferation of each scaffold could provide a feasible environment for the cells, the relative absorption between the third and seventh day was significantly different. However, the relative absorption between each kinds of scaffold was not statistically significant which demonstrated that the ICG-001 was not toxic to the epithelial cells (Figure 6).

### 2.5. Immunofluorescence of Anti-Fibrosis Effect of Medium Released from Scaffold

**Figure 7:**
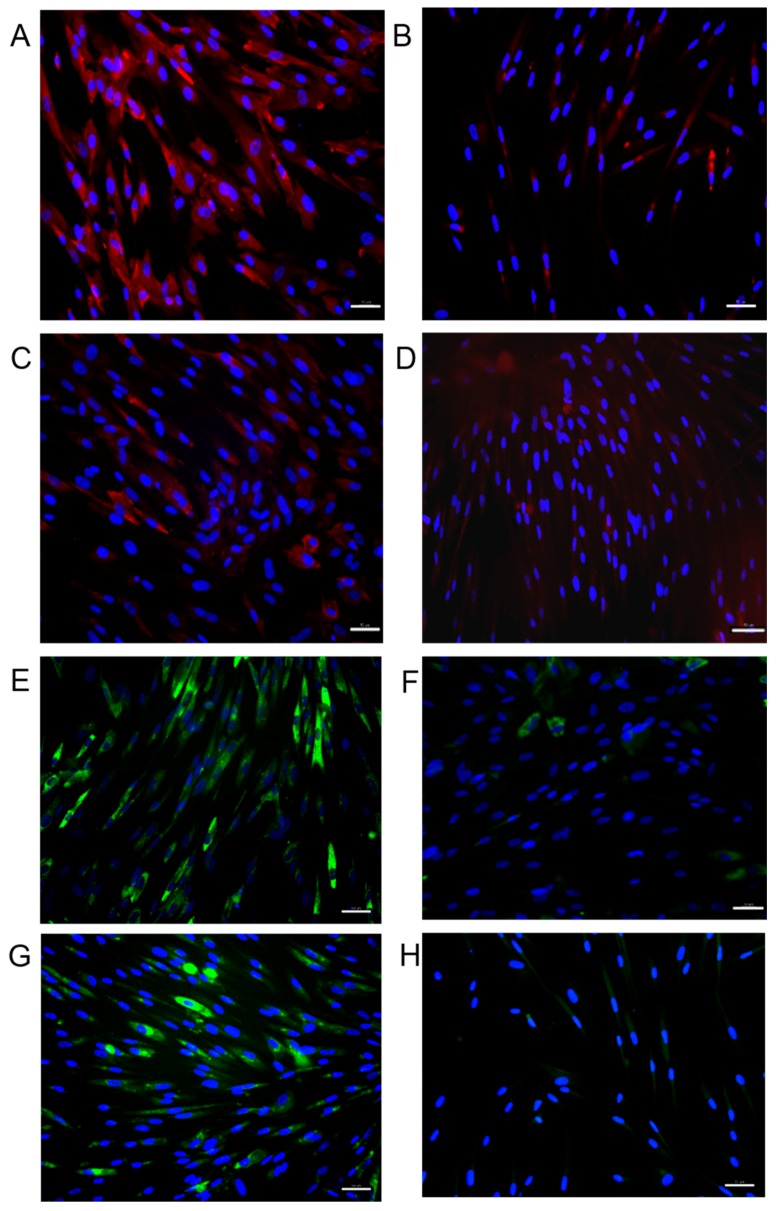
The collagen type 1 expression of fibroblasts in immunofluorescence (Red): ((**A**) TGF-β1 treated; (**B**) Treated with both TGF-β1 and culture medium from Col/P(LLA-CL)/ICG-001; (**C**) Untreated fibroblasts and (**D**) Non-TGF-β1 treated fibroblasts with treatment of culture medium from Col/P(LLA-CL)/ICG-001). The collagen type 3 expression of fibroblasts in immunofluorescence (Green): ((**E**) TGF-β1 treated; (**F**) Treated with Both TGF-β1 and culture medium from Col/P(LLA-CL)/ICG-001; (**G**) Untreated fibroblasts and (**H**) Non-TGF-β1 treated fibroblasts with treatment of culture medium from Col/P(LLA-CL)/ICG-001). Scale bar: 50 µm. Blue: Nuclei.

Fibroblasts were treated with TGF-β1 with or without ICG-001 for three days to evaluate the phenotype change of myofibroblasts and ECM expression. The signals of collagen type 1 and 3 was significantly reinforced in the TGF-β1 treated fibroblasts (Figure 7A,E). Collagen type 1 and 3 expression levels were decreased in the fibroblasts treated with ICG-001 in TGF-β1 treated fibroblasts (Figure 7B,F). The fibroblasts without TGF-β1 treatment (Figure 7C,G) also showed decreased collagen type 1 and 3 expression levels after treatment of ICG-001 released medium (Figure 7D,F).

### 2.6. Real Time PCR

**Figure 8:**
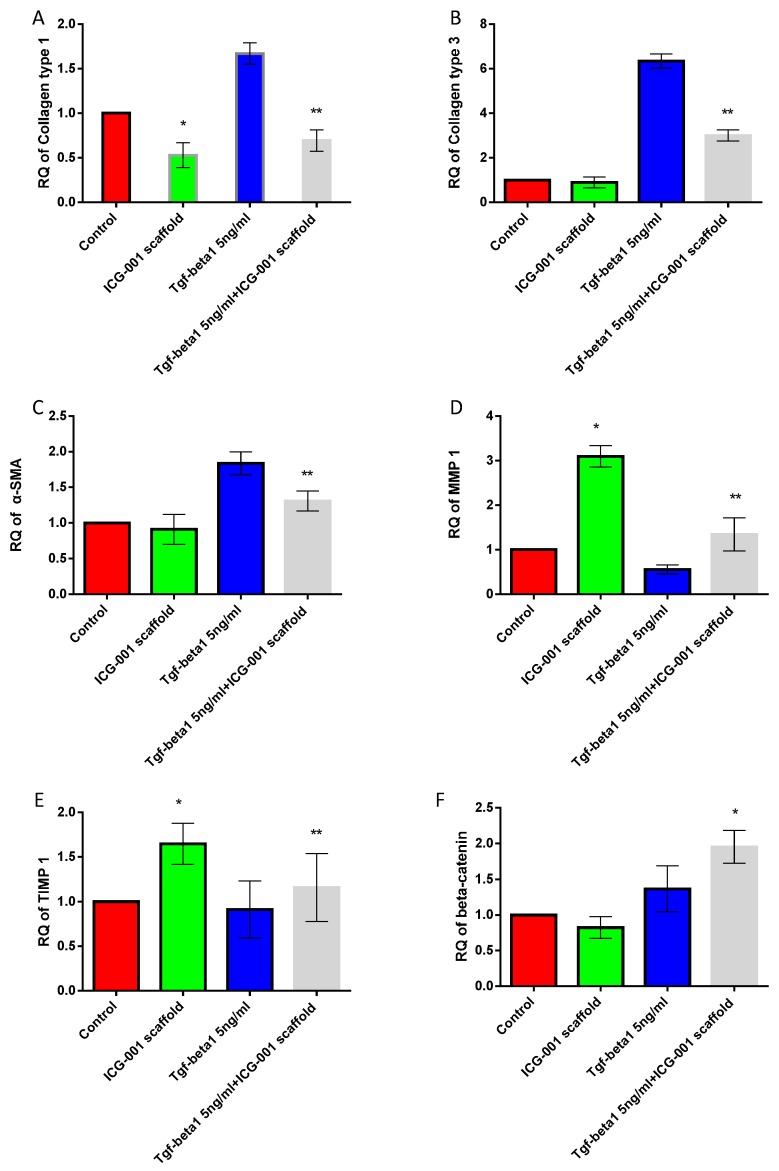
mRNA expression levels of the real-time PCR detection of collagen type 1 (**A**); collagen type 3 (**B**); α-smooth muscle actin (α-SMA) (**C**); Matrix metalloproteinase 1 (MMP1) (**D**); Tissue inhibitor of metalloproteinases 1 (TIMP1) (**E**) and β-catenin (**F**) under different conditions. * *p* < 0.05 compared with control group; ** *p* < 0.05 compared with TGF-β1 treated group.

Quantitative real-time PCR was performed at day three to evaluate the change of gene expression of fibroblasts at the mRNA level (Figure 8). Collagen type 1 (Figure 8A), collagen type 3 (Figure 8B), α-smooth muscle actin (α-SMA) (Figure 8C), Matrix metalloproteinase 1 (MMP1) (Figure 8D), Tissue inhibitor of metalloproteinases 1 (TIMP1) (Figure 8E) and β-catenin (Figure 8F) were analyzed for fibroblasts treated with TGF-β1 or not. Compared with the TGF-β1 treatment group, the ICG-001 culture medium could decrease the expression of collagen type 1, 3 and α-SMA significantly and elevate the mRNA expression of MMP1 and TIMP1 significantly. The ICG-001 medium could decrease mRNA expression of collagen type 1 and elevate the MMP1 and TIMP1 significantly compared with untreated fibroblasts.

### 2.7. Western Blot

**Figure 9:**
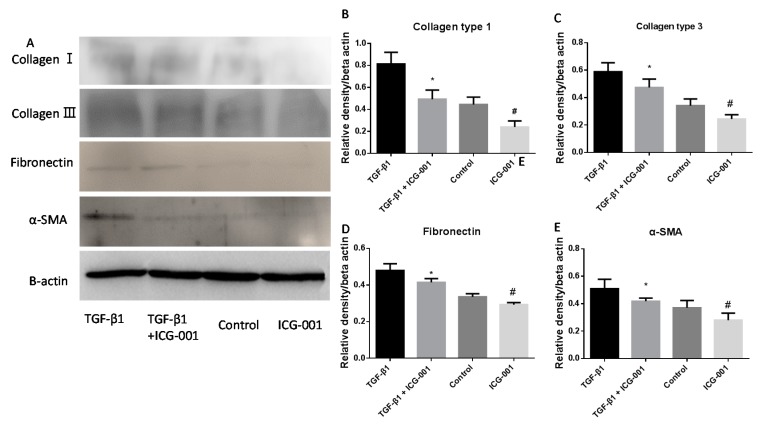
Western blot (**A**) and relative expression levels of fibrosis related proteins (**B**–**E**): Collagen type 1 (**B**), collagen type 3 (**C**), fibronectin (**D**) and α-SMA (**E**) in fibroblasts treated with TGF-β1 and culture medium released from ICG-001/scaffold determined by western blot analysis. * *p* < 0.05 compared with TGF-β1 group; # *p* < 0.05 compared with control group.

Western blot was performed for relative quantitative analysis of collagen type 1, collagen type 3, fibronectin and α-SMA (Figure 9A). The quantitative results were consistent with the immunofluorescence staining and real time PCR, the relative expression of collagen type 1 (Figure 9B), collagen type 3 (Figure 9C), fibronectin (Figure 9D) and α-SMA (Figure 9E) to β-actin decreased with the adding of culture medium of ICG-001 delivering scaffold, especially in the groups of TGF-β1 treated fibroblasts.

### 2.8. Urethrography and Surgery Outcomes

**Figure 10:**
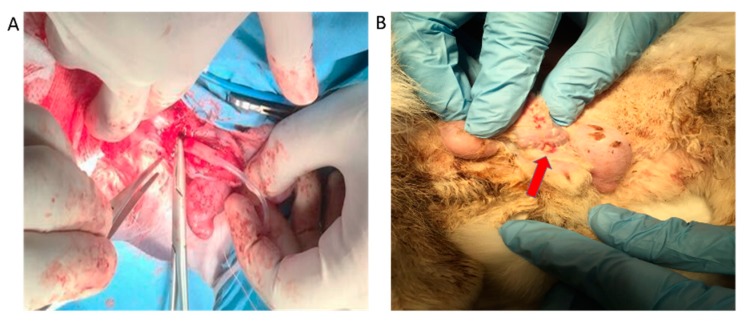

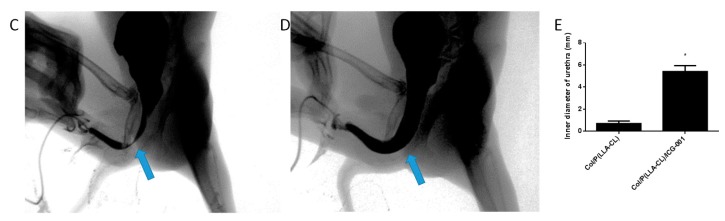
The surgery process and complication. The tubularized scaffolds were implanted into the urethral defects (**A**); A fistula developed at the penile skin in group 1 (**B**). The red arrow indicates the position of fistula; The representative image of retrograde urethrography after rabbit urethroplasty with the non-drug scaffold (**C**); and ICG-001 delivering scaffold (**D**); the lumen diameter of urethras are shown in (**E**). The blue arrow indicates the surgery position. * *p* < 0.05.

Twelve rabbits in two groups underwent operations and survived within three months. Five out of six rabbits in group 1 developed narrow urethral lumens according to the urethrography (Figure 10C) and one rabbit developed a fistula at the penile skin (Figure 10B). All the rabbits in group 2 showed unrestricted lumen (Figure 10D) and no sign of fistula at the penile skin was found. The results demonstrated that epithelial cell seeded Col/P(LLA-CL) scaffolds were successfully used in the reconstruction of 2 cm urethral defects in rabbit models.

### 2.9. Histology and Immunohistology Results

**Figure 11:**
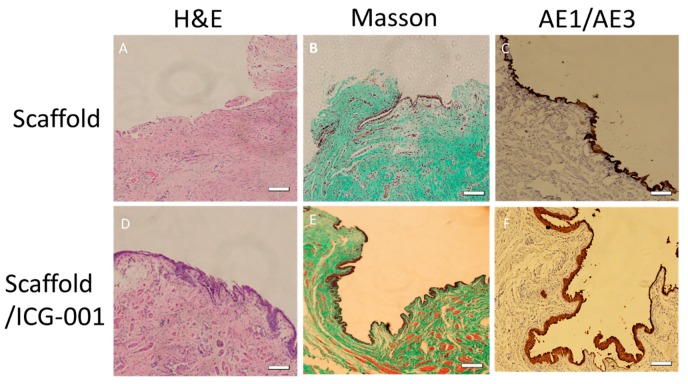

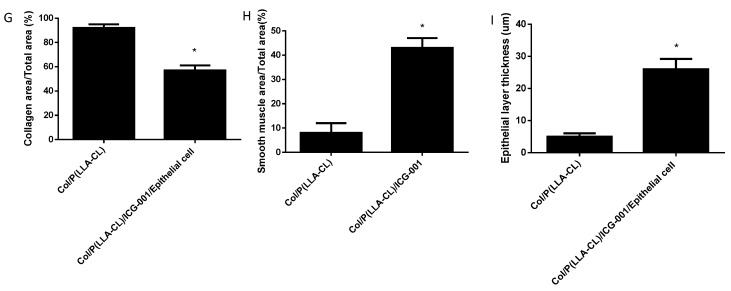
Hematoxylin and eosin stain (H&E) staining of repaired urethra with non-drug scaffold (**A**) and ICG-001 delivering scaffold (**D**); Masson staining of repaired urethra with non-drug scaffold (**B**) and ICG-001 delivering scaffold (**E**); and AE1/A E3 staining of repaired urethra with non-drug scaffold (**C**) and ICG-001 delivering scaffold (**F**) at three months postoperatively. Scale bar = 100 µm; The quantitative analysis of the histology showed the relative collagen area (**G**); relative smooth muscle area (**H**); and epithelium thickness (**I**); *n* = 10. * *p* < 0.05.

In histology test of group 1, the lumen surface formed discontinued epithelial layer according to the H&E staining (Figure 11A) and AE1/AE3 (Figure 11C) immunohistology image. The tissue in the urethra showed a large amount of collagen and less smooth muscle according to the Masson staining image (Figure 11B). However, in group 2, the epithelial cells developed multiple layer epithelium (Figure 11D,F). The tissue in the submucosa developed more smooth muscle and less collagen in the Masson image (Figure 11E). The quantitative analysis with image J showed the significant difference of collagen (Figure 11G), smooth muscle (Figure 11H) and epithelium (Figure 11I) between the two groups.

## 3. Discussion

In the present study, it was demonstrated that the biocompatibility and mechanical properties of the ICG-001-delivering collagen/P(LLA-CL) electrospun scaffold was sufficient for bladder epithelial cells proliferation and could be applied to urethroplasty. The co-axial electrospun scaffold delivering ICG-001 delivered drug release in a controlled fashion and had obvious effects of anti-fibrosis.

The ideal scaffolds could undertake many tasks such as to provide the necessary mechanical property and deliver inductive biomolecules [37,38]. Scaffolds made of nanofibrous materials with electrospinning approach have been applied for tissue engineering, both for replacement and regeneration [33,39]. Recently, great efforts have been made in the field of biomaterials in order to discover new methods to deliver therapeutic agents through the co-axial electrospinning technique [40,41]. Release of molecules from scaffolds can significantly improve the scaffold ability of directing tissue regeneration *in vitro* and *in vivo* [42,43]. As well known, gene techniques are an efficient approach to influence cell destiny, however, such genetic technology is very difficult to apply to patients due to ethical and technical aspects [44]. Therefore, biomaterials for delivering signaling pathway inhibitors have a higher attraction for surgeons in the clinic.

The advantage of core-shell co-axial electrospinning secured a controlled release of nearly 75% ICG-001 in scaffolds as long as 30 days. According to the instruction of the manufacturer, the pharmaceutical activity of ICG-001 could be maintained three months at room temperature. It was reported that the urethral scar formed in the first month after urethral injury [45,46], so the pharmaceutical activity could be sufficient for reducing scar formation for the *in vivo* study in the future.

Wnt signaling has been implicated into the pathogenesis of various human disorders such as fibrotic diseases [47]. Activation of canonical Wnt signaling induces fibroblast activation with excessive ECM release, resulting in tissue fibrosis that disrupts the normal physiological tissue architecture [48]. Beyer *et al.* demonstrated that the use of small molecule drugs to inhibit the Wnt pathway is an effective method for the treatment of fibrosis; it could obtain a similar effect of inhibiting the TGF-β pathway, but has good cell tolerance [26]. In the SEM and MTT assay for epithelial cells on scaffolds, the proliferation rate was not influenced significantly by ICG-001, which demonstrated that the biocompatibility of ICG-001 is acceptable. Many studies on the molecular mechanisms of Wnt signaling pathway inhibitor have been performed; however, to our knowledge, there is no previous report about the application of Wnt signaling interference with regenerative medicine. We have used the culture medium released from the ICG-001 delivering scaffold to inhibit the ECM expression of fibroblasts *in vitro*. In the immunofluorescence studies, highest expression of collagen type 1 and 3 was noticed in the TGF-β1 treated group; when combined with ICG-001-released medium, the expression showed an obvious decline. This phenomenon was also found in the group with ICG-001-released medium alone compared with the control group.

Real time PCR revealed the effects of ICG-001 at the RNA level. RNA expression of collagen type 1, collagen type 3, and α-SMA was significantly elevated in the groups with ICG-001-released medium compared with the TGF-β1 alone, and control group. α-SMA is a biomarker of myofibroblasts which demonstrated the phenotype change of fibroblasts and higher expression of the ECM gene [49]. Myofibroblasts are considered as the main cause of scar formation in many fibrosis related disease [50]. The RNA expression of both MMP1 and TIMP1 were elevated when ICG-001 was applied, the balance of both genes are important for scar formation. Although TIMP1 elevated with MMP1, the ultimate ECM genes expression was down regulated.

Western blot results demonstrated that the ICG-001-released solution could inhibit the production of ECM related protein at the protein level, including α-SMA, collagen type 1, 3 and fibronectin. The intensity of western blot bands in the ICG-001 group was decreased significantly compared with the TGF-β1 treated group and control group respectively. The results were consistent with that in the fluorescence and real-time PCR.

Based on *in vitro* study, we investigated the therapeutic effect of the ICG-001 delivering scaffold in the rabbit urethra defect model. Collagen deposition was inhibited significantly by the ICG-001 delivering scaffold according to the histology. ICG-001 could be released from the scaffold gradually after transplanting into the urethral defect to inhibit the inflammation and ECM deposition during the healing process of the urethra. In our previous meta-analysis about anti-fibrosis drugs for urethral stricture, the efficacy of various drugs were not yet verified [51]. This functional ICG-001 delivering scaffold might become a candidate for preventing the urethra from recurrent stricture.

It was reported that the airway epithelial cell layer could be maintained by ICG-001 and that airway epithelial cell apoptosis was significantly decreased in bleomycin-induced animal models [29]. This character of ICG-001 might play an important role in urethral epithelial cells, thus besides the anti-fibrosis effect, the epithelial layer in experimental group was thicker than the control group. The limitation of the present study is that a more appropriate animal model is needed to evaluate the treatment outcomes of post-traumatic urethral strictures.

## 4. Experimental Section

Poly(l-lactide-*co*-caprolactone) (P(LLA-CL)) (LA:CL = 50:50, *M*_W_ = 300,000) was provided by Nara medical university. Type I collagen was obtained from Sichuan Ming-Rang Bio-Tech Co., Ltd. (Chengdu, Sichuan, China). And 2,2,2-trifluoroethanol was from Fine chemicals, Shanghai, China. The ratio of collagen: P(LLA-CL) = 25:75. ICG-001 was purchased from Selleckchem (Houston, TX, USA).

### 4.1. Fabrication of Nanofibrous Scaffold Delivering ICG-001

Collagen/P(LLA-CL) scaffolds delivering ICG-001 (Figure 1) were constructed using a co-axial electrospinning device (Donghua University, Shanghai). The solution of the core layer was 1 g collagen/P(LLA-CL) dissolved in 2, 2, 2-trifluoroethanol; then it was mixed with 1 mg ICG-001 in 60 µL DMSO solution and injected at a rate of 0.2 mL/h. The solution of the shell layer was 1 g Collagen/P(LLA-CL) dissolved in 2, 2, 2-trifluoroethanol and fed at 0.8 mL/h. During the process of scaffold fabrication, room temperature was kept within 22–25 °C, and the relative humidity was 40%–50%. A stainless dish was used to collect the nanofibers. The distance between the sprayer tip and the receiving dish was set to 15 cm and the positive voltage was 18 kV. The scaffolds were kept under vacuum at room temperature for 48 h before being used.

The non-drug co-axial collagen/P(LLA-CL) electrospun nanofiber scaffold was fabricated as a control group, all the processes were same, but 60 µL DMSO without ICG-001 was added in the core solution.

### 4.2. Scanning Electron Microscopy of Scaffolds

Scanning electron microscopy (SEM, Hitachi TM-100, Tokyo, Japan) was used to observe the morphology of the scaffolds. Specimens were punched into 1.2 cm-diameter disks and cryopreserved at −80 °C for 2 h, then freeze-dried overnight and preserved in a vacuum container. The specimens of scaffolds were imaged under SEM. Nanofiber diameter was measured with 200 fibers by image analysis software Image-J.

### 4.3. Mechanical Property Evaluation

To compare the mechanical properties of different scaffolds, small intestinal submucosa (SIS), a commercial biomaterial for urethra reconstruction, was used as the control material. Tensile strength was measured by an instron tensile tester (model 5544; Norwood, MA, USA). All samples were prepared as longitudinal strips (20 mm in length and 10 mm in width). Each sample of scaffolds was fixed onto the clamps and pulled at 5 mm/minute crosshead speed until rupture. Burst pressure was measured by gradually increasing hydrostatic pressure within the scaffolds at a rate of 80 mmHg/min. Then Bluehill software (Norwood, MA, USA) was used to calculate the maximal tensile strength.

### 4.4. In Vitro Release Test with High-Performance Liquid Chromatography

The ICG-001 delivering scaffold was punched into dishes with 1.2 cm in diameter and weighed; the weight was 60 mg. They were then placed into 1.5 mL eppendorf tubes. Each tube was filled with 1 mL phosphate buffered saline (PBS) solution and sealed tightly, and then incubated in an air rotator at 100 rpm in 37 °C. At the initial time point and predetermined time intervals, 100 µL of the supernatant in the tubes was collected and fresh PBS in equal volume was added. The release of ICG-001 in the buffer was detected by high-performance liquid chromatography (HPLC) in the Biomaterials and Tissue Engineering Laboratory of Donghua University, China. The eluent used in the HPLC process is acetonitrile (Jinjingle Chemical Engineering, Shanghai, China). A standard curve was made with gradient dilution of the ICG-001 DMSO solution from 10 to 10 thousand. The releasing experiments were performed and compared with the standard curve in triplicate every three days and completed until 30 days. The data were obtained and carefully analyzed to determine the concentration of ICG-001 released from the specimens at each immersion time point.

### 4.5. Cell Isolation and Identification

All the animal experiments were in accordance with the guidelines for animal care. The animal protocol (SYXK 2011-0128) was approved by the animal ethics committee of Shanghai Sixth People’s Hospital, Shanghai, China. The project identification code is 14JC1492100, which is approved from 1 September 2014 by Science and technology commission of Shanghai, China.

Bladder biopsies and epithelial cell harvesting were made using 12 male New Zealand white rabbits. The rabbits were pretreated with 15 mg/kg Ketamine, 2 to 3 mg/kg xylazine and 0.75 mg/kg acepromazine intramuscularly, then they were anesthetized and maintained with 2% isoflurane. A small laparotomy incision was made above the pubic symphysis to expose the bladder. A biopsy specimen with 2 × 2 cm was excised from the bladder wall, then the defect was closed with 3-0 polyglactin sutures in 2 layers. The rabbits were given 5 mg/kg enrofloxacin intramuscularly for 3 days after operation.

The specimen was processed in a sterile condition. It was washed with PBS with 100 IU/mL penicillin and 100 µg/mL streptomycin. The epithelium was scraped from smooth muscle layer after using dispase type 2 enzyme (Roche) at 4 °C overnight. Then the epithelium was cut into small fractions and incubated in 0.25% trypsin solution for 15–30 min. The solution of cells were collected and cultured in 10 cm culture dish coated with 1% Type 1 rat tail collagen. Defined keratinocyte serum-free medium (DKSFM, Life Technologies, Carlsbad, CA, USA) with supplements was used as culture medium. Before being seeded into the scaffold, epithelial cells were identified with anti-pan cytokeratin antibody AE1/AE3 (ab27988; Abcam, Cambridge, UK).

Dermal fibroblasts were used to perform the collagen inhibition assay in this study. Two cm^2^ of rabbit dermal tissue at abdomen was excised from one rabbit and rinsed under sterile conditions, then it was cut into small pieces and digested with collagenase type 1. The cells were cultured in 6-well plates with DMEM supplemented with 10% fetal calf serum. Then they were identified with vimentin antibody before being used (Santa Cruz, Dallas, TX, USA).

### 4.6. In Vitro Analysis of Epithelial Cell-Seeded Scaffolds

To prevent waste of ICG-001 in the sterilizing process with 70% ethanol, the scaffolds were sterilized with ultraviolet for 2 h. The epithelial cells were seeded on the surface of the scaffolds with 5 × 10^5^ cells/cm^2^. Cells were cultured with defined keratinocyte serum free medium (DKSFM) for 7 days before being used.

At days 3 and 7, cell-seeded scaffolds were rinsed with PBS to remove the non-adherent cells. Then the cells with scaffolds were fixed in 2.5% glutaraldehyde for 30 min at room temperature. Afterwards, they were dehydrated through a series of graded alcohol solutions. The drying process was conducted with the critical point dryer (Donghua university, Shanghai). The scaffolds were sputter coated with gold-palladiu (AuPd), and examined under SEM at 12 kV.

### 4.7. MTT Assay

Cell proliferation was tested quantitatively by using the MTT assay at day 1, 3 and 7. Cells on scaffold were incubated with MTT (5 mg/mL in DMEM without phenol red; Sigma-Aldrich, St. Louis, MO, USA). After 3 h of incubation, the medium was transferred into wells of a 96-well plate. The data were read at 490 nm in a synergy plate reader.

### 4.8. Fibroblast Induction and Solution Preparation

Ten thousand fibroblasts were transferred to each well of 4-well chamber slides and cultured overnight. The ICG-001 delivered scaffold was sterilized with ultraviolet for 2 h. To collect the ICG-001 medium, 2 mL of complete culture medium was used to immerse 120 mg ICG-001 delivered scaffold to get ICG-001 solution for 24 h. TGF-β1 (Life Technologies) 5 ng/mL was used to induce a phenotype change from fibroblasts to myofibroblasts and overexpress ECM according to the protocol [52]. Four groups were set in the study with adding TGF-β1, ICG-001 solution alone or simultaneously to fibroblasts. Group 1: TGF-β1; Group 2: TGF-β1 + ICG-001 solution; Group 3: untreated fibroblasts; and Group 4: ICG-001 solution. Three days after culturing, the fibroblasts were used in various tests.

### 4.9. Immunofluorescence

The primary monoclonal antibodies were anti-collagen type 1 and anti-collagen type 3 from mouse (Sigma, St. Louis, MO, USA). The cells were treated with 0.2% Triton X-100 for 10 min at room temperature and incubated with the primary antibody for 60 min at 37 °C, then the cells were rinsed with PBS 3 times and incubated with primary antibody and fluorescent labeled secondary antibody (Donkey anti-Mouse IgG (heavy + light chain) Secondary Antibody, Alexa Fluor^®^ 488 and 594 conjugate, Thermo Fisher Scientific, Waltham, MA, Country) for 30 min at 37 °C. The nuclei were stained with Fluoroshield Mounting Medium (Sigma, St. Louis, MO, USA) with DAPI. The cells were examined with fluorescence microscopy.

### 4.10. RNA Extraction and Real Time PCR

At day 3 of treating, total RNA was extracted for the quantification of collagen type 1, type 3, TIMPs, MMPs, β-catenin and α-SMA (Rneasy maxi kit, Qiagen, Valencia, Spain), cDNA was synthesized (TaqMan RT, Roche Molecular Biochemicals, Indianapolis, IN ,USA). TaqMan probes were applied for the quantification of the target genes. β-Actin was used as an endogenous quality control.

### 4.11. Western Blot Analysis

At day 3, western blot analysis was conducted to analyze the relative expression level of collagen type 1, 3, fibronectin and α-SMA in fibroblasts treated with TGF-β1 and culture medium released from the ICG-001 delivering scaffold. The lysis buffer (Radio-Immunoprecipitation Assay buffer and Phenylmethanesulfonyl fluoride, Thermal Scientific, Waltham, MA, USA) was used to extract the proteins. After running on a 6% gel, proteins were transferred to nitrocellulose membranes (Bio-rad, Hercules, CA, USA). Membranes were blocked with Tris-buffered saline with 0.1% Tween-20 (TBST) containing 5% nonfat dry milk at room temperature, then incubated with primary antibodies at 4 °C overnight and subsequently with HRP (horseradish peroxidase)-conjugated goat anti-mouse secondary antibody for 1 h at room temperature. Anti-β-actin antibody was used as a protein loading control. The results were quantified using Quantity one and showed as the relative expression to β-actin.

### 4.12. Rabbit Urethroplasty

Twelve male New Zealand white rabbits were divided into 2 groups. Six rabbits were in group 1 and treated with non-drug scaffold with epithelial cells seeded. Group 2 were treated with an ICG-001 delivering scaffold with epithelial cells seeded. After general anesthesia with intravenous injection of pentobarbital, Foley F8 silicone catheters (Suzhou, Jiangsu, China) were inserted into the urethra of 18 male rabbits. All surgeries were performed by an urologist. Briefly, the skin approximately 3 cm from the external urethral orifice was sectioned, and the urethra was dissected from the corpus cavernosum. Ventral urethral defects (mean length of 2.0 cm and width of 0.8 cm) were created in the bulbar urethra of rabbits. The scaffolds (length of 2 cm and width of 1 cm) were tubularized and sutured to form tubes, then sutured to the urethra defect with 6-0 absorbable polyglactin sutures. The 8F silicone catheter was left in the urethra and fixed to the glan of the rabbit with 6-0 absorbable sutures for 14 days postoperatively. The animals were observed twice on each day before catheters were removed; if the animal removed the catheter, another new catheter would be reinserted after anesthesia. Euthanization of the rabbits in the 2 groups after 3 months was planned.

### 4.13. Urethrography

Retrograde urethrograms were made for the animals in both groups to assess urethral caliber before animals were euthanized.

### 4.14. Histology and Immunohistology Assessment

The urethras were harvested for histology analysis. Hematoxylin and eosin stain (H&E), Masson trichrome staining and AE1/AE3 immunohistology test were conducted to identify the epithelium layer, smooth muscle and collagen. The Masson staining images were used to collagen and smooth muscle analysis, and the AE1/AE3 staining images were used for epithelial analysis.

### 4.15. Statistical Analysis

Results are expressed as mean ± standard deviation. SPSS statistical software 16.0 (Chicago, IL, USA) was applied to calculate the data by one-way analysis of variance; *p* < 0.05 was considered statistically significant.

## 5. Conclusions

We successfully constructed ICG-001 delivering collagen/P(LLA-CL) scaffolds with high mechanical properties. The *in vitro* study verified the biocompatibility and long term release fashion of ICG-001 in the scaffold. Immunofluorescence, PCR and Western blot results demonstrated that the ICG-001 delivering scaffolds are able to significantly inhibit ECM expression of fibroblasts. The results presented here using the rabbit urethral defect model provides a foundation for further study with potential clinical applications in the future.
